# Diffuse cranial vault hyperostosis associated with long‐term antiepileptic therapy: Autopsy findings and differential diagnostic considerations

**DOI:** 10.1111/1556-4029.70416

**Published:** 2026-07-16

**Authors:** Maria Piagkou, Athina Tousia, George Triantafyllou, Dimitrios Vlachodimitropoulos, Samer Al Besher, Panagiotis Manolarakis‐Papadopoulos, Alexandros Samolis, Stavroula Papadodima

**Affiliations:** ^1^ Department of Anatomy, Faculty of Health Sciences, School of Medicine National and Kapodistrian University of Athens Athens Greece; ^2^ Department of Forensic Medicine and Toxicology, School of Medicine National and Kapodistrian University of Athens Athens Greece; ^3^ Department of Neurosurgery General Hospital of Nikaia‐Piraeus Nikaia Greece

**Keywords:** antiepileptic drugs, autopsy, calvarial thickening, cranial vault hyperostosis, forensic pathology, phenytoin

## Abstract

Diffuse cranial vault hyperostosis is an uncommon finding and may present a diagnostic challenge in clinical and forensic practice. We report the case of a 53‐year‐old woman with a long‐standing history of epilepsy treated with phenytoin and sodium valproate who collapsed at home suddenly and died despite resuscitative efforts. Post‐mortem examination revealed marked, symmetrical thickening of the frontal, parietal, and occipital bones, with cranial vault thickness reaching approximately 20 mm. Histological analysis demonstrated expansion of the diploë with preserved lamellar architecture and absence of marrow hyperplasia, while examination of the dura mater revealed focal fibrosis and ossification. No evidence of trauma, neoplasia, metabolic bone disease, or hematologic disorder was identified. In the absence of alternative etiologies and in the context of prolonged antiepileptic drug exposure, a medication‐related cranial remodeling process was considered the most plausible explanation, most plausibly associated with chronic phenytoin exposure. Death was attributed to recent myocardial ischemia in the setting of pneumonia and cerebral atrophy. This case underscores the importance of integrated clinicopathological correlation when evaluating unusual cranial thickening and highlights the forensic relevance of recognizing medication‐associated skeletal remodeling to avoid diagnostic misinterpretation.

**Highlights**

- Diffuse cranial vault hyperostosis is an uncommon incidental postmortem finding.
- Chronic antiepileptic therapy should be considered in the differential diagnosis of diffuse cranial hyperostosis.
- Histology can help exclude Paget disease, neoplastic infiltration, and other possible causes.
- Recognition of this entity may prevent misinterpretation of skull findings.

## 1 INTRODUCTION

Physiological cranial vault thickness varies according to sex, age, and anatomical region, but generally does not exceed 10–12 mm in adults [1]. Cranial vault thickening beyond these limits is uncommon and warrants careful evaluation for underlying pathology. The differential diagnosis is broad and includes endocranial hyperostoses such as hyperostosis frontalis interna (HFI), metabolic disorders (e.g., Paget disease of bone, osteopetrosis, fibrous dysplasia), endocrine abnormalities (e.g., acromegaly and hyperparathyroidism), hematologic conditions associated with marrow expansion, and neoplastic processes, including hyperostotic meningioma en plaque and calvarial metastases [2, 3, 4, 5, 6].

Although these entities account for most cases of pathological cranial thickening, medication‐related alterations in bone metabolism should also be considered as an additional, often underrecognized cause. Long‐term antiepileptic drug (AED) therapy, particularly with phenytoin and valproate, has been associated with disturbances in calcium and vitamin D homeostasis, altered osteoblastic activity, and dysregulated bone remodeling [7]. Although the skeletal effects of antiepileptic medication are most often discussed in relation to osteopenia, osteoporosis, reduced bone mineral density, fracture risk, and other abnormalities of bone metabolism, the literature also includes less common reports of calvarial thickening and diffuse cranial hyperostosis associated with chronic anticonvulsant exposure, especially with phenytoin [8, 9]. The cranial vault may be especially susceptible to such changes, likely reflecting lifelong remodeling activity and the regulatory influence of the dura mater on calvarial osteogenesis [8, 9].

Diffuse cranial vault hyperostosis, however, remains a rare and insufficiently characterized manifestation within this spectrum. In contrast to classical HFI, which is typically confined to the inner table of the frontal bone and spares the midline, diffuse forms involving the frontal, parietal, and occipital bones pose significant diagnostic challenges and may overlap with other pathological entities. In forensic and autopsy settings, failure to recognize medication‐related skeletal remodeling may lead to misinterpretation, including erroneous attribution to trauma, systemic bone disease, or occult malignancy.

Here, we present a rare case of diffuse cranial vault hyperostosis associated with long‐term AED therapy, accompanied by marked alveolar hyperostosis. By integrating gross anatomical, histopathological, and clinical data, this study aims to refine the differential diagnosis, explore potential pathophysiological mechanisms—particularly the role of phenytoin‐induced bone remodeling—and highlight the forensic implications of this underrecognized condition. To our knowledge, such comprehensive clinicopathological correlation remains limited in the current literature.

## 2 CASE PRESENTATION

A 53‐year‐old woman with a longstanding history of epilepsy, managed with chronic antiepileptic medication including phenytoin and valproate, died suddenly and unexpectedly. No ante‐mortem cranial imaging was available, and post‐mortem computed tomography (CT) was not performed.

At autopsy, marked and symmetrical thickening of the cranial vault was identified (Figures 1B, 2A). The frontal, parietal, and occipital bones were diffusely involved, with maximal vault thickness approaching 20 mm. By comparison, normal adult cranial vault thickness typically ranges from 5 to 7 mm in the parietal region and 6–8 mm in the frontal bone, with upper limits rarely exceeding 10–12 mm [1]. The thickening was uniform, with smooth inner and outer tables and a markedly expanded diploic space (Figure 1). No macroscopic evidence of cranial trauma, infection, neoplasia, or generalized skeletal disease was observed. In addition, examination of the oral cavity demonstrated an edentulous maxilla with marked enlargement of the alveolar ridge and diffuse fibrous gingival soft‐tissue overgrowth (Figure 2A), findings consistent with longstanding drug‐related hyperplastic changes and associated alveolar osseous remodeling. External examination also revealed a healed surgical scar over the left trochanteric and gluteal region, consistent with prior orthopedic intervention. The available history included a previous hip fracture.

**FIGURE 1:**
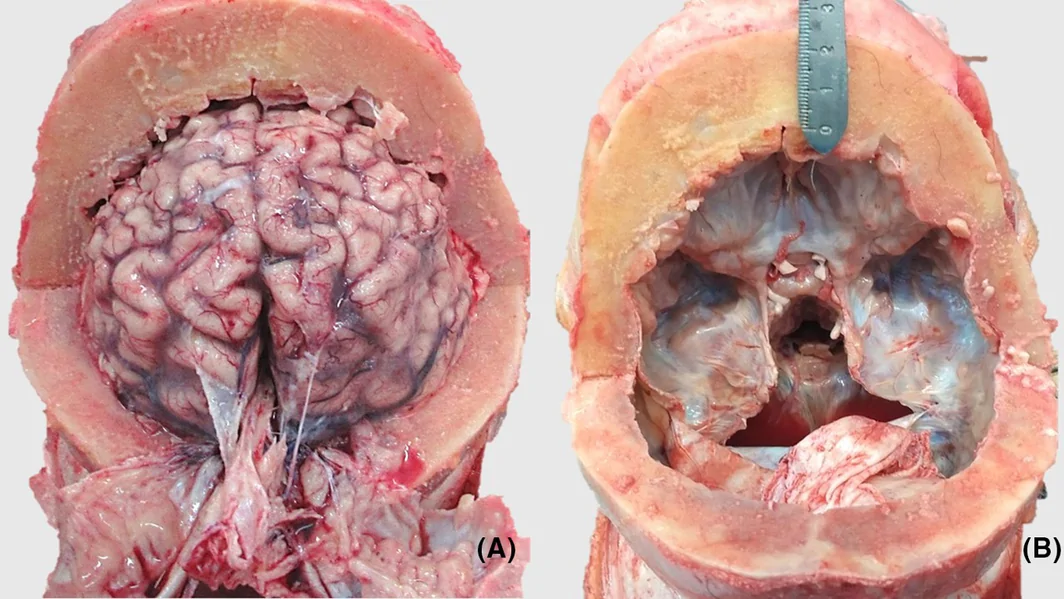
Diffuse cranial vault hyperostosis identified at post‐mortem examination. (A) Superior view of the opened cranial vault with the brain in situ, demonstrating marked circumferential thickening of the frontal, parietal, and occipital bones, resulting in a rigid cranial ring and relative reduction of intracranial space. The inner table appears smooth and continuous, without focal erosions or nodular excrescences. (B) Internal view of the calvarial ring after brain removal. The inner table and expanded diploë are uniformly thickened, with preservation of cortical contours and absence of focal lesions. A metallic ruler highlights the increased thickness of the cranial vault, which reaches approximately 20 mm.

**FIGURE 2:**
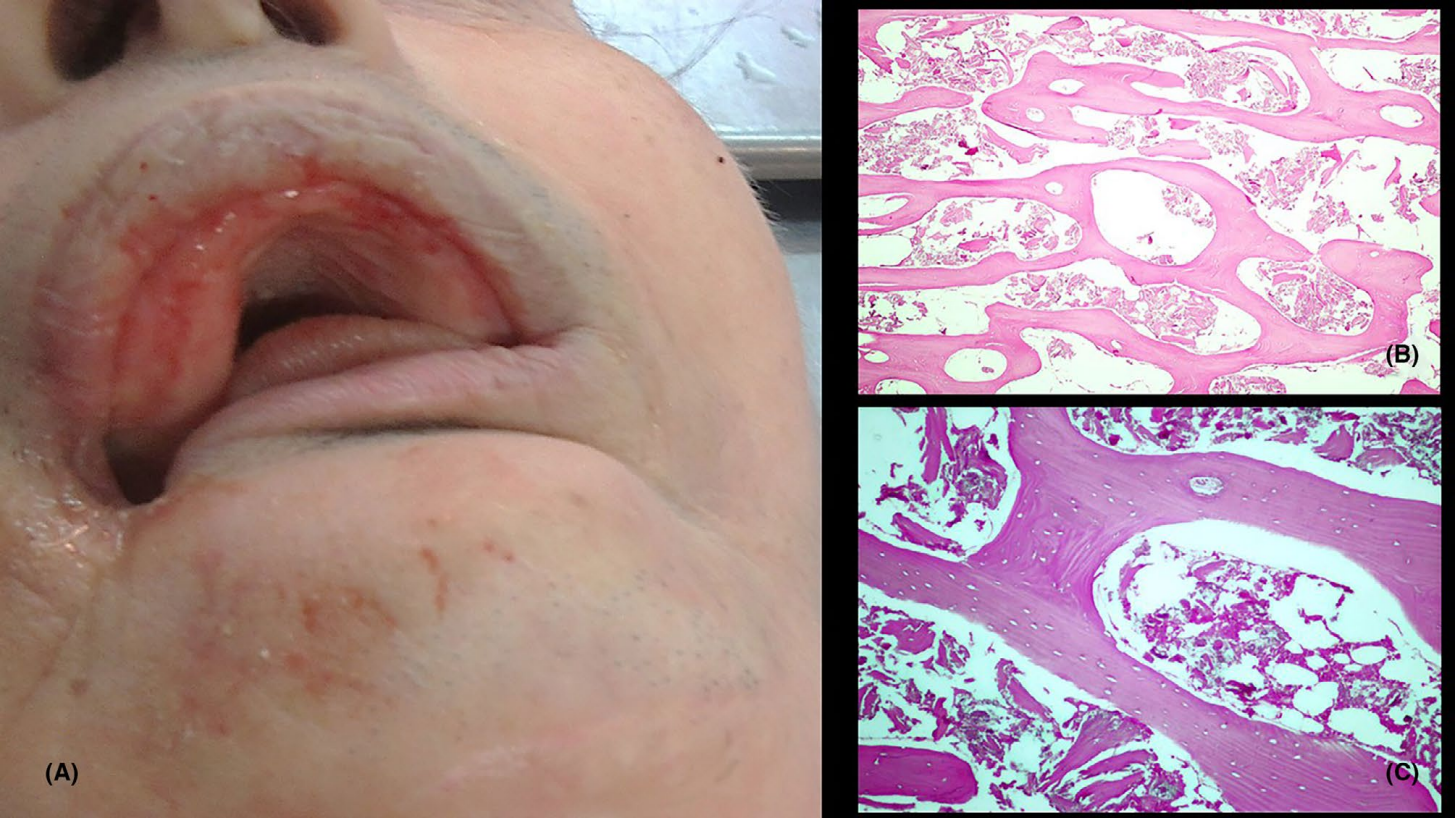
(A) Craniofacial manifestation associated with chronic antiepileptic therapy. Intraoral view of the edentulous maxilla demonstrating marked enlargement of the alveolar ridge with diffuse gingival soft‐tissue overgrowth. The prominent fibrous mucogingival thickening is consistent with longstanding drug‐related hyperplastic changes and associated alveolar osseous remodeling. (B) Histological section of the cranial vault (hematoxylin and eosin stain, low magnification) demonstrating expansion of the diploic compartment with thickened trabeculae of mature lamellar bone and preserved overall architecture. Marrow spaces contain predominantly fatty marrow. No mosaic remodeling, woven bone formation, marrow hyperplasia, or neoplastic infiltration is identified. (C) Histological section of the cranial vault (hematoxylin and eosin stain, higher magnification) showing well‐organized mature lamellar trabecular bone with preserved osteocyte lacunae and smooth remodeling surfaces. Marrow spaces contain predominantly fatty marrow. No increased osteoclastic activity, fibro‐osseous change, woven bone formation, mosaic patterning, or malignant cellular infiltration is identified.

Histological examination of calvarial sections demonstrated pronounced expansion of the diploë with preservation of normal lamellar bone architecture. There was no evidence of marrow hyperplasia, woven bone formation, or mosaic lamellar patterning (Figure 2B,C). Examination of the dura mater revealed focal fibrosis, hyalinization, and areas of ossification. The brain was reduced in weight (760 g) but showed no specific histopathological abnormalities (Figure 1A). Biochemical parameters related to bone metabolism, including serum calcium, vitamin D, parathyroid hormone, and bone turnover markers, were unavailable.

Given the absence of morphological and histological features suggestive of metabolic, genetic, neoplastic, or hematologic disease, and in the context of prolonged antiepileptic drug exposure, the findings were considered most consistent with diffuse cranial vault hyperostosis. A medication‐related remodeling process plausibly associated with chronic phenytoin exposure was regarded as the most likely underlying mechanism. The cause of death was certified as recent myocardial ischemia in the setting of pneumonia and cerebral atrophy, with a history of epilepsy. The manner of death was classified as natural.

## 3 DISCUSSION

Diffuse cranial vault hyperostosis represents a heterogeneous condition with a broad differential diagnosis, and its interpretation requires careful correlation of gross morphology, histopathology, clinical history, and pharmacological exposure. Among benign hyperostotic disorders, HFI is the most frequently encountered entity, predominantly affecting postmenopausal women and typically confined to the inner table of the frontal bone [10, 11]. Although HFI may share certain histological features with the present case, its characteristic restriction to the frontal region does not account for the diffuse involvement of the parietal and occipital bones observed here. While unusually extensive variants within the spectrum of endocranial hyperostosis have been described, the circumferential distribution and marked diploic expansion in the present case favor a more generalized remodeling process rather than a typical HFI variant [12]. Furthermore, the relative sparing of the cranial base, without endosteal thickening or narrowing of cranial foramina, argues strongly against hyperostosis cranialis interna, in which basicranial involvement and progressive foraminal stenosis are defining features [13].

Other metabolic and systemic conditions were also considered. Paget disease of bone is characterized by disorganized remodeling with mosaic lamellar architecture and frequently asymmetric cranial involvement, often accompanied by elevated biochemical markers of bone turnover [4]; none of these features were identified. Osteopetrosis, which presents diffuse skeletal sclerosis and obliteration of marrow spaces along with systemic hematologic and neurological manifestations, was not supported by the findings [5]. Similarly, fibrous dysplasia typically demonstrates fibro‐osseous replacement and woven bone formation and presents earlier in life; features that were absent in this case [14].

Ex vacuo cranial hyperostosis should also be considered, as compensatory cranial thickening has been described in association with cerebral atrophy and intracranial volume loss. Similar findings have been reported following severe cerebral injury sustained early in life and in Dyke‐Davidoff‐Masson syndrome [15, 16]. Although reduced brain weight was present in this case, the marked diploic expansion and preserved cortical architecture favor a primary remodeling process rather than a purely compensatory mechanism.

Metabolic and endocrine disorders such as renal osteodystrophy with secondary hyperparathyroidism and acromegaly also form part of the differential diagnosis. However, the available clinical and pathological data did not support these entities. There were no reported phenotypic features suggestive of acromegaly, nor evidence of advanced chronic kidney disease or generalized metabolic bone disease. Associations between endocranial hyperostosis and endocrine disturbances such as hypoparathyroidism have also been described [17].

Neoplastic causes, particularly hyperostotic meningioma en plaque, may produce cranial thickening that mimics benign hyperostosis; however, such lesions are typically focal or asymmetric and associated with dural masses or histological evidence of tumor infiltration [18]. In contrast, the preserved lamellar architecture, absence of marrow hyperplasia, and symmetrical distribution observed in the present case support a chronic remodeling process rather than an infiltrative or aggressive pathology.

In this context, long‐term antiepileptic drug (AED) therapy provides a plausible mechanistic explanation. The skeletal consequences of chronic AED exposure are more commonly described in relation to osteopenia, osteoporosis, reduced bone mineral density, and increased fracture risk. Nevertheless, less common reports have documented calvarial thickening and diffuse cranial hyperostosis in patients receiving prolonged anticonvulsant therapy, particularly phenytoin [7, 8, 9]. Among AEDs, phenytoin has been consistently associated with diffuse calvarial thickening, primarily due to diploic expansion with preservation of the inner and outer cortical tables, a pattern first described radiologically by Kattan in 1970 [8]. Subsequent studies have confirmed this vault‐predominant phenotype and attributed it to osteoblastic stimulation mediated by growth‐factor signaling pathways [9]. In parallel, valproate has been linked mainly to reduced bone mineral density and altered osteoblast function, suggesting a more modulatory role in bone metabolism [7].

The morphology observed in the present case—namely diffuse, symmetrical calvarial thickening with marked expansion of the diploë and preservation of cortical architecture—closely mirrors the characteristic pattern of phenytoin‐associated cranial remodeling. The concomitant alveolar ridge enlargement and gingival overgrowth further support chronic phenytoin‐related remodeling of the craniofacial skeleton and soft tissues. In addition to direct osteoblastic activation, anticonvulsant therapy may promote calvarial hyperostosis through secondary hyperparathyroidism related to altered vitamin D metabolism, with parathyroid hormone exerting divergent skeletal effects that favor bone apposition in the calvarium [19].

Furthermore, the presence of focal dural fibrosis and ossification supports the concept of a chronic dura‐mediated remodeling process. Experimental studies have shown that the dura mater is not merely a passive membrane but an active regulator of calvarial osteogenesis. In animal models, the dura contributed substantially to new bone formation, while regional differences in dural osteoinductive potential were also demonstrated [20, 21]. Recent developmental studies further support that the dura regulates cranial morphogenesis and osteogenic differentiation through local signaling pathways [20]. Experimental cranial defect models have additionally demonstrated differential regenerative responses between dural and periosteal surfaces [21]. These findings may help explain the preferential localization of thickening to the cranial vault and the relative sparing of the cranial base in the present case.

From a forensic perspective, diffuse cranial vault thickening may complicate the interpretation of cranial trauma by altering fracture biomechanics or obscuring fracture patterns. Importantly, despite the marked degree of hyperostosis, no intracranial hemorrhage, herniation, or mass effect was identified, arguing against a direct contribution to death. The certified cause of death was recent myocardial ischemia in the setting of pneumonia and cerebral atrophy, with a history of epilepsy, supporting the interpretation that the cranial finding was incidental rather than lethal. The reduced brain weight observed at autopsy is more plausibly related to chronic epilepsy and/or long‐term antiepileptic therapy, while prolonged phenytoin exposure has also been associated with chronic neurological adverse effects in selected patients [22]. Accordingly, the cranial hyperostosis in this case should be regarded as an incidental but contextually significant anatomical finding rather than a primary cause of death.

This study has several limitations. The absence of antemortem imaging and postmortem CT precluded detailed radiological assessment of the cranial vault and its potential effects on intracranial volume and cranial foramina. Additionally, biochemical parameters related to bone metabolism, including serum calcium, vitamin D, parathyroid hormone, and bone turnover markers, were unavailable, limiting the evaluation of metabolic contributors. As a single case report, a causal relationship between AED therapy and cranial hyperostosis cannot be definitively established. Furthermore, detailed data regarding cumulative drug exposure, duration of therapy, and potential drug interactions were not available. Finally, the lack of longitudinal clinical follow‐up precludes assessment of disease progression and functional implications.

## 4 CONCLUSION

Diffuse cranial vault hyperostosis appears to be a rare and likely underrecognized skeletal manifestation associated with long‐term antiepileptic therapy. Accurate interpretation requires integration of pharmacological history with detailed anatomical and histopathological assessment. Recognition of medication‐related cranial remodeling is important in clinical and forensic practice, particularly at autopsy, where unusual skull thickening may otherwise raise concern for trauma, neoplasia, or systemic bone disease.

## CONFLICT OF INTEREST STATEMENT

The authors have no conflicts of interest.

## Data Availability

The data that support the findings of this study are available from the corresponding author upon reasonable request.
